# Case report: Intraneural perineurioma of the sciatic nerve in an adolescent – strategies for revealing the diagnosis

**DOI:** 10.1002/ccr3.630

**Published:** 2016-07-06

**Authors:** Lars B. Dahlin, Inger Nennesmo, Jack Besjakov, Istvan Ferencz, Gert S. Andersson, Clas Backman

**Affiliations:** ^1^ Department of Translational Medicine – Hand SurgerySkåne University HospitalLund UniversityMalmöSweden; ^2^ Department of PathologyKarolinska University HospitalStockholmSweden; ^3^ Medical Radiology UnitSkåne University HospitalLund UniversityMalmöSweden; ^4^ Department of Clinical Sciences in Lund – NeurologyLund UniversityLundSweden; ^5^ Department of Clinical Sciences in Lund – NeurophysiologyLund UniversityLundSweden; ^6^ Department of Surgical and Perioperative ScienceUniversity HospitalUmeåSweden

**Keywords:** Imaging, immunohistochemistry, nerve tumors, neuropathology, targeted fascicular nerve biopsy

## Abstract

Diagnosis of intraneural conditions can be revealed by a combination of clinical examination, electrophysiology, magnetic resonance imaging (MRI), and targeted fascicular biopsy with subsequent microscopic analyses.

## Introduction

Nerve tumors are rare. The most common nerve tumors are neurofibroma and Schwannoma; the latter is usually treated with enucleation and rarely with remaining symptoms or recurrence 1, 2. However, some pathological conditions with tumor formation may be difficult to reveal, since they develop insidiously in young patients and cause predominantly motor dysfunction with milder sensory involvement. An intraneural perineurioma is a condition that may be underrecognized due to slow progression and sometimes low morbidity 3, but with a clinical suspicion, an adequate imaging technique, targeted fascicular biopsy of the lesion, and evaluation by an experienced neuropathologist the diagnosis can be made. Intraneural perineurioma may develop in a major nerve trunk, particularly in the sciatic nerve 3, where magnetic resonance imaging (MRI) shows focal nerve enlargement with contrast enhancement 4. A targeted fascicular nerve biopsy of the identified focal lesion is advisable to get a proper diagnosis 5. The intraneural perineurioma has a characteristic microscopic feature with pseudo‐onion bulb formations of the perineurial cells with reactivity for epithelial membrane antigen (EMA) and S‐100 reactivity only confined to the centrally located myelinated nerve fibers 6, 7. Here, we report on a patient with a long history of insidiously developing symptoms, particularly motor involvement and muscle atrophy, affecting the sciatic nerve, where the diagnosis was revealed by a targeted fascicular nerve biopsy and a detailed microscopical evaluation.

## Case Report

**Figure 1:**
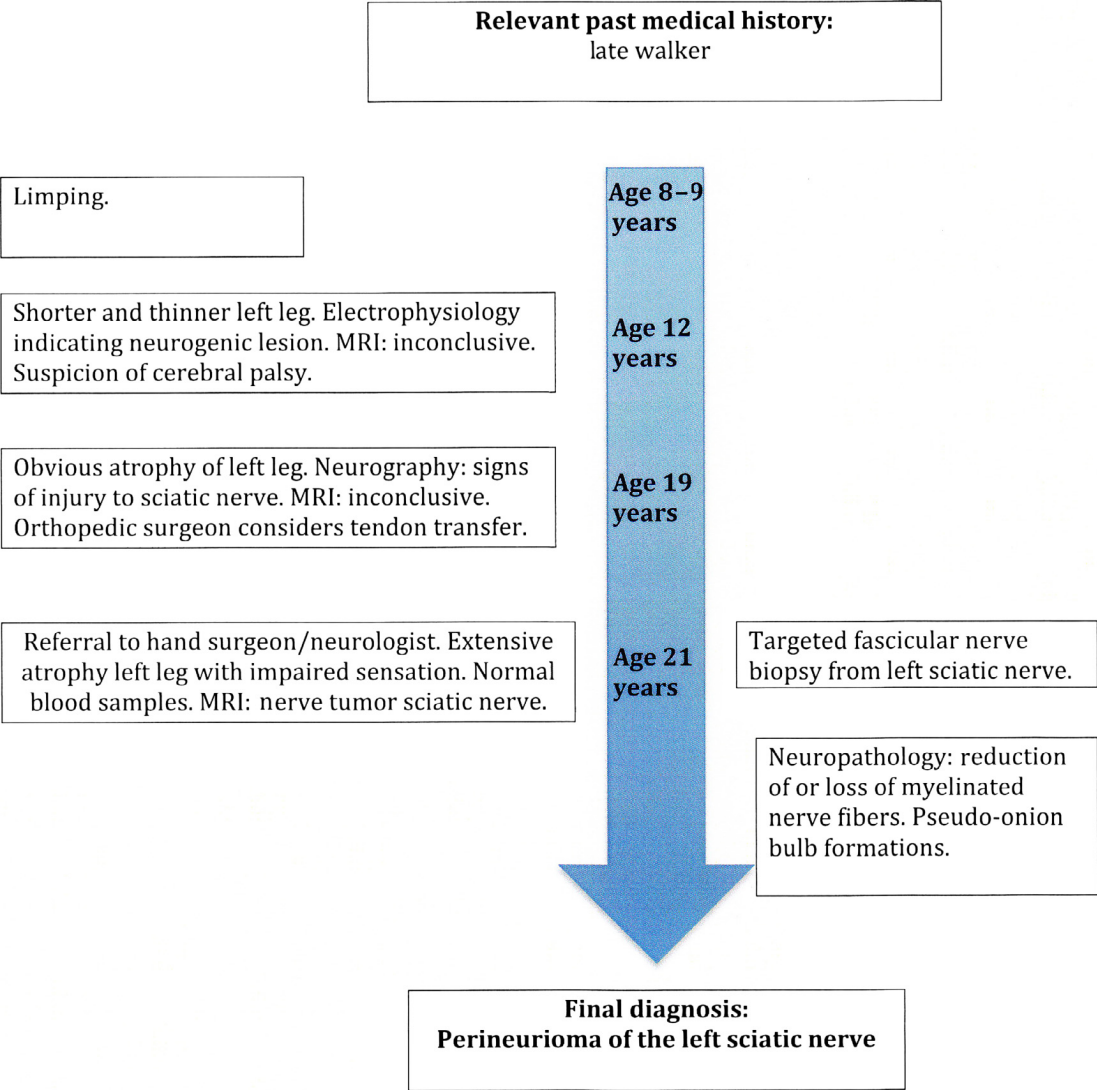
Timeline.

The patient was referred to Department of Hand Surgery at the age of 21 due to weakness in the left leg, particularly below knee level (Fig. 1). He has a twin and learned to walk later than his younger sister (at 1.5 years and at 1 year, respectively). From the age of 8–9, his parents noticed that he limped, particularly in sporting activities. He was first examined in a nearby hospital at the age of 12. The left leg was 1 cm shorter than the right one, thinner, and the circumference of the thigh and lower leg was 1 cm shorter than on the contralateral side. An electrophysiological examination showed slow motor conduction velocities and low amplitudes in the tibial and peroneal nerves as well as an absent sensory response from the left sural nerve, while the findings in the right leg and from the left median nerve were completely normal. Electromyography showed severely neurogenic rebuilt motor units in the anterior tibial and gastrocnemius muscles on the left side. There was also ongoing denervation activity. The conclusion was that there was a neurogenic lesion affecting the peroneal and the tibial nerves in the left leg. A MRI of the lumbar spine showed that the left S1 root was without any fat layers, particularly between the nerve and the disk, but no other findings were observed. Initially, there was a suspicion of a cerebral palsy. The patient saw a physiotherapist on a regular basis and had follow‐up visits with orthopedic surgeons concerning the length of the leg and a pes planovalgus. A serial casting was tried due to the short Achilles tendon, which had developed due to the weakness of the muscles in the anterior muscle group in the leg. Later, no differences in length of the lower extremities were noted. However, an extensive atrophy of the muscles in the lower left leg was observed. The patient experienced a slight foot drop. During the process, the patient developed more pain at load and pain from several joints.

Clinical examination by a child neurologist when the patient was 19 showed obvious atrophy of not only peroneal innervated muscles but also of the gastrocnemius and soleus muscles without any visible fasciculations. The sensation was normal down to knee level, but more distally, there was impaired sensation with almost complete lack of sensation in the toes. The dorsal extension was absent and the plantar flexion was markedly impaired. Reflexes were normal except that the left Achilles reflex was very weak. The neurography and EMG showed signs of injury to the left sciatic nerve proximally at thigh level, probably distal to the branch to the semimembranosus and semitendinosus muscles and proximally to the branch innervating the short head of the biceps femoris muscle (thus, the peroneal part of the sciatic nerve). There was a clear progress of axonal degeneration since the previous examination. A MRI of the left thigh was inconclusive.

**Figure 2:**
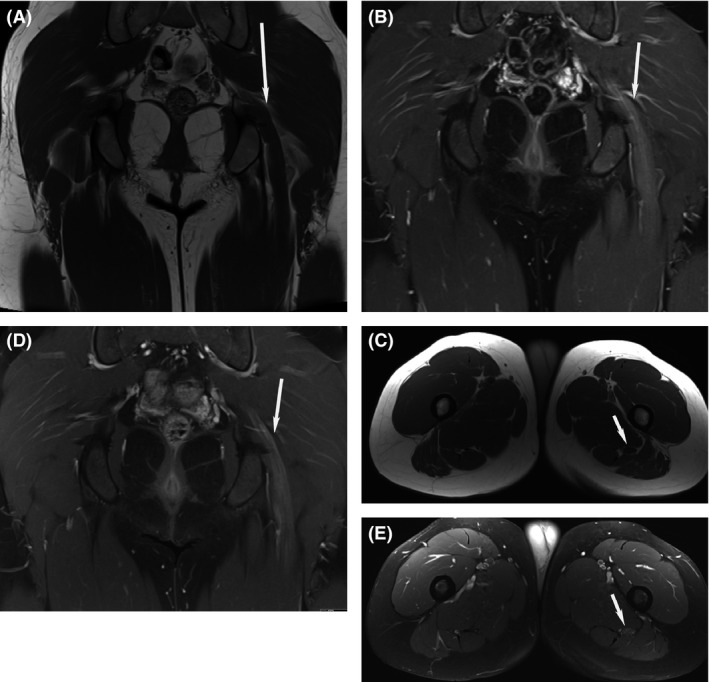
Magnetic resonance imaging (MRI) using coronal T1‐weighted turbo spin echo (TSE) (A), short‐tau inversion recovery (B), and axial T1‐weighted TSE (C) images were obtained. Following gadolinium administration, coronal and axial T1‐weighted fat‐suppressed TSE were obtained (D, E). The perineurioma is marked with arrows.

The patient was referred to an orthopedic surgeon, who considered doing a tendon transfer to improve dorsal extension. Due to a suspicion of nerve affection, he was referred to a peripheral nerve unit (hand surgeon and neurologist). At examination, there was impaired sensation particularly from knee level and distally except the innervation area of the saphenous nerve. No tendon reflexes were found in the left leg (normal in the right). The quadriceps and hamstrings had good strength, but the semimembranosus muscle did not have the same volume as on the contralateral right side. There was an extensive atrophy of the distal leg, but he could still activate some parts of the peroneal muscles and the most lateral toe extensors. Pulses were normal. Blood samples were also normal. Imaging using 1.5‐Tesla MRI was obtained before and after intravenous administration of gadolinium in coronal as well as in axial projections of the pelvis and both thighs (Fig. 2A–E) showing a nerve tumor. The left sciatic nerve demonstrated changes from the acetabular roof level to about 11 cm above the knee joint, which corresponds to a length of 40 cm. Distal to the pathologic changed left sciatic nerve, there were no detectable changes visible on the MRI sequences with and without intravenous gadolinium administration.

**Figure 3:**
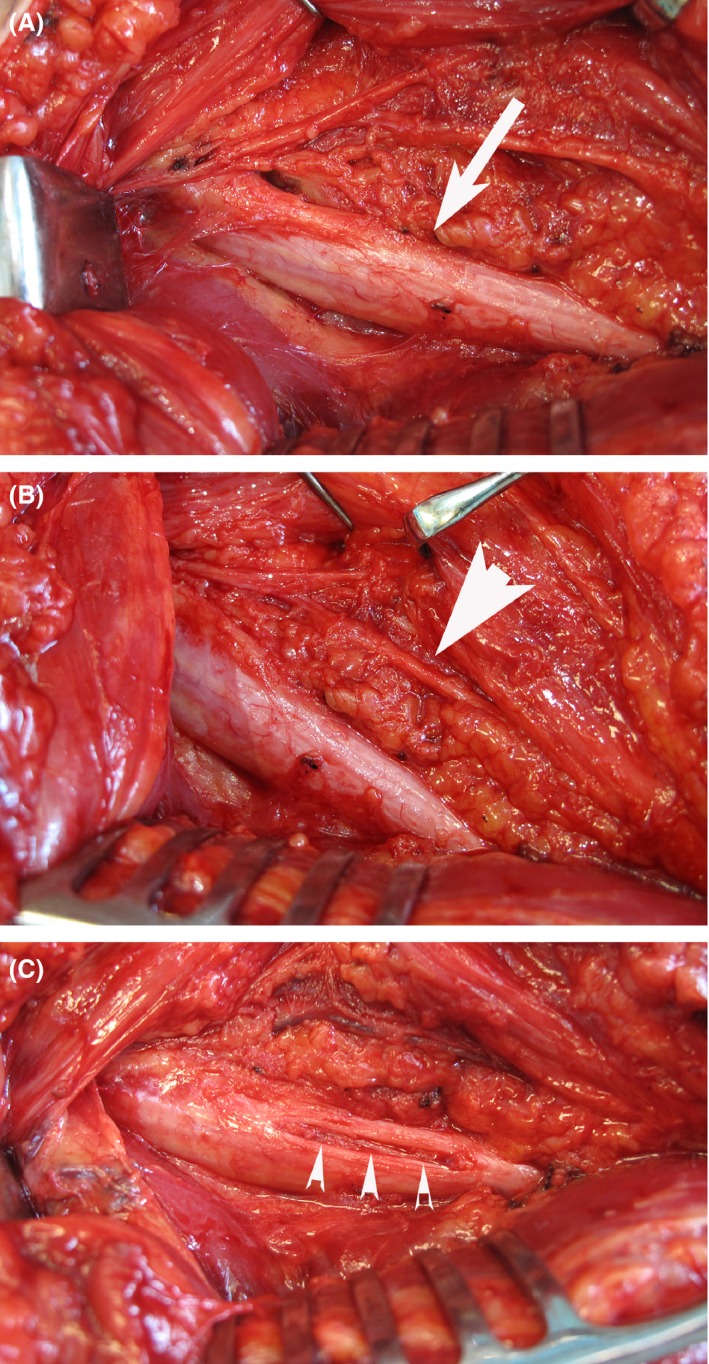
Peroperative photos with the enlarged sciatic nerve (arrow; A) and a muscular branch (arrowhead, B), which were biopsied. The site of the fascicular biopsy in the sciatic nerve (C) is indicated by three arrowheads.

Since there was a suspicion of an unknown affection of the most proximal part of the sciatic nerve, a targeted fascicular biopsy from the tumor (Fig. 3A) was performed when the patient was 22. At surgery, three biopsies were taken: (i) motor branch to semimembranosus muscle (Fig. 3B); (ii) sensory branch to the distal part of the thigh; (iii) fascicular biopsy from posterior part of the main trunk of the sciatic nerve (Fig. 3C).

### Pathological examination

A nerve branch to the semimembranosus muscle (1.8 cm long), a sensory branch of the sciatic nerve (2.2 cm long), and an intraneural sciatic fascicle from the tibial component (0.9 cm long) were fixed in glutaraldehyde and then placed in a buffer. After arrival to the neuropathology laboratory, they were post fixed in glutaraldehyde for three days, and thereafter processed for paraffin and plastic embedding. On paraffin sections, Htx–eosin, Luxol fast blue, Congo red, and iron staining as well as immunostaining with antibodies against CD68 were performed for all biopsies. On the tibial component, immunohistochemistry with antibodies against S‐100 and epithelial membrane antigen (EMA) was also made.

**Figure 4:**
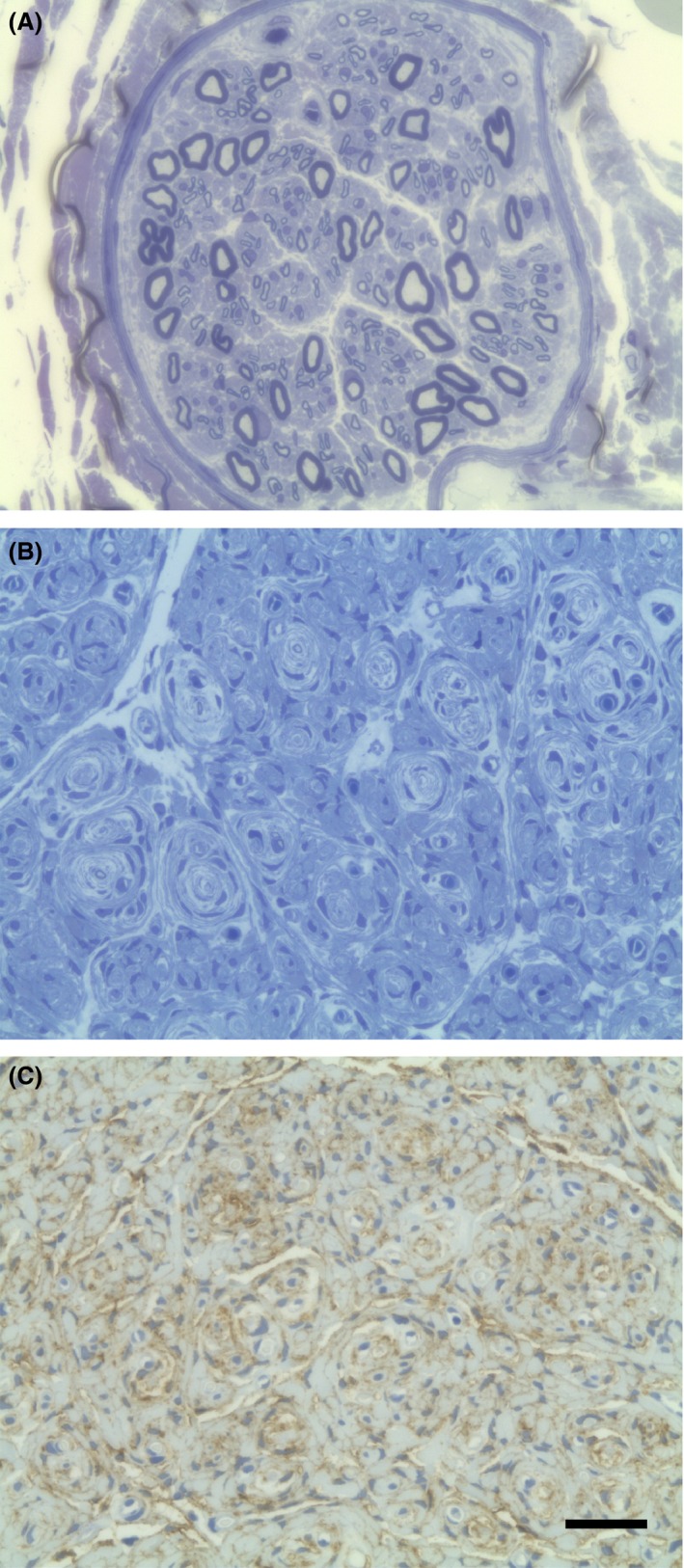
Light microscopy from the sensory nerve branch (A) with a slight reduction in nerve fibers and from the tibial component of the sciatic nerve (B, C) showing no normal nerve fibers and only pseudo‐onion bulb formations, where EMA staining (C) was positive only in the pseudo‐onion bulbs. A 1 *μ*m section from Epon‐embedded specimens with toluidine blue staining (A and B) and EMA staining from paraffin‐embedded sections (C). Length of bar = 20 *μ*m.

The branch to the semimembranosus muscle and the sensory branch contained three small and nine small to medium‐sized fascicles, respectively. There was a slight reduction in myelinated fibers (Fig. 4A). The tibial component consisted of one large fascicle in which no normal myelinated fibers could be seen; only pseudo‐onion bulb formations were present (Fig. 4B). Immunostaining with S‐100 demonstrated reactivity only at the center of these, while EMA showed positivity only in the surrounding pseudo‐onion bulb leaflets (Fig. 4C). In electron microscopy, concentrically arranged cellular processes around thinly myelinated axons were found.

## Discussion

The present case report represents a strategy on how to investigate a peripheral nerve problem with impaired function irrespective of location 3, 4. There was a long‐standing history of impaired function; an early sign was the delayed walking ability compared to the twin sister. Particularly at the age of 8–9, at sports activities, the patient noticed impaired function and limping. At the age of 12, there were already signs of affection of the muscles of the lower leg and this was particularly obvious at the final examination at the age of 21, where the EMG and ENG showed clear signs of sciatic nerve lesion proximally at thigh level. Based on that information and a thorough clinical examination, excluding other causes of neuropathy, a detailed MRI visualized the affected sciatic nerve 4. The level of the intraneural perineurioma could be defined, which allowed a targeted fascicular biopsy; a procedure that did not add any residual problems 5. Interestingly, few findings were found in the two branches leaving the main sciatic nerve trunk; essentially, no signs of intraneural perineurioma per se, only some loss of myelinated fibers. More importantly, the fascicles that were harvested from the main sciatic nerve trunk, after detailed electrophysiological stimulation thus avoiding fascicles with functional axons and thereby not further impairing the function of the nerve, revealed the definitive diagnosis, that is, intraneural perineurioma with pseudo‐onion bulbs formations and typical immunostaining pattern (Fig. 4A–C).

We conclude that in patients with a single affected nerve, infrequently affecting muscles in a patchy pattern, a detailed history has to been taken, and a thorough clinical examination is essential. Based on the detailed information from electrophysiology and MRI, it is possible to perform a targeted fascicular biopsy 5 which should be adequately stained, in the present case with antibodies against S‐100 and epithelial membrane antigen (EMA; 6). Thus, the diagnosis can be made properly. Even if the condition cannot be treated, as in intraneural perineurioma, it is important for the patient to get a proper diagnosis and hopefully an indication of the prognosis. The patient is presently just having clinical follow‐ups and is provided with suitable orthosis (foot drop), since there is no specific procedure to treat this type of nerve tumor.

## Conflict of Interest

None declared.
